# Supplementary material for: Contextual associations represented both in neural networks and human behavior
Source: Sci Rep. 2022 Apr 2;12:5570. doi: 10.1038/s41598-022-09451-y (PMC8976848; doi:10.1038/s41598-022-09451-y)

Contextual associations represented both in neural networks and human behavior

Elissa M. Aminoff, Shira Baror, Eric W. Roginek, & Daniel D. Leeds

SUPPLEMENTAL MATERAL

Supplemental Figure: Each graph represents the similarity ratio for category (orange) and context (blue) in each of the networks tested. All networks show a significant effect of contextual associations.

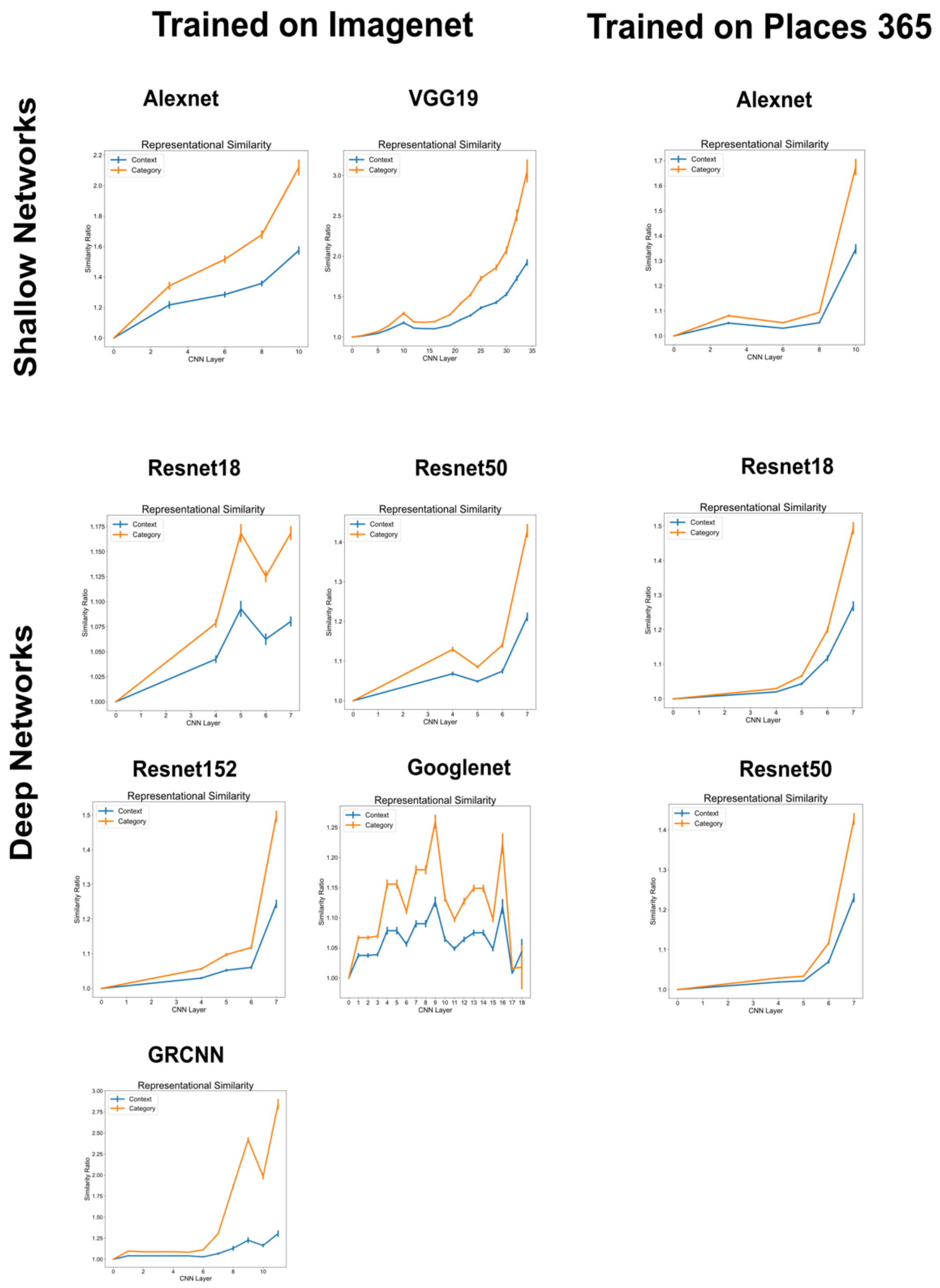

Supplement: Supplementary file 1 — Supplementary Figure 1. [file 41598_2022_9451_MOESM1_ESM.pdf]
